# Study of excess manganese stress response highlights the central role of manganese exporter Mnx for holding manganese homeostasis in the cyanobacterium Synechocystis sp. PCC 6803

**DOI:** 10.1099/mic.0.001515

**Published:** 2024-11-07

**Authors:** Mara Reis, Sanja Zenker, Prisca Viehöver, Karsten Niehaus, Andrea Bräutigam, Marion Eisenhut

**Affiliations:** 1 Computational Biology, Center for Biotechnology (CeBiTec) and Faculty of Biology, Bielefeld University, Bielefeld, Germany; 2 Genetics and Genomics of Plants, Center for Biotechnology (CeBiTec) and Faculty of Biology, Bielefeld University, Bielefeld, Germany; 3 Proteome and Metabolome Research, Center for Biotechnology (CeBiTec) and Faculty of Biology, Bielefeld University, Bielefeld, Germany

**Keywords:** cyanobacteria, manganese, regulation, RNA-seq, toxicity, transporter

## Abstract

Cellular levels of the essential micronutrient manganese (Mn) need to be carefully balanced within narrow borders. In cyanobacteria, a sufficient Mn supply is critical for ensuring the function of the oxygen-evolving complex as the central part of the photosynthetic machinery. However, Mn accumulation is fatal for the cells. The reason for the observed cytotoxicity is unclear. To understand the causality behind Mn toxicity in cyanobacteria, we investigated the impact of excess Mn on physiology and global gene expression in the model organism *Synechocystis* sp. PCC 6803. We compared the response of the WT and the knock-out mutant in the *Mn* e*x*porter (Mnx), ∆*mnx*, which is disabled in the export of surplus Mn and thus functions as a model for toxic Mn overaccumulation. While growth and pigment accumulation in ∆*mnx* were severely impaired 24 h after the addition of tenfold Mn, the WT was not affected and thus mounted an adequate transcriptional response. RNA-seq data analysis revealed that the Mn stress transcriptomes partly resembled an iron limitation transcriptome. However, the expression of iron limitation signature genes *isiABDC* was not affected by the Mn treatment, indicating that Mn excess is not accompanied by iron limitation in *Synechocystis*. We suggest that the ferric uptake regulator, Fur, gets partially mismetallated under Mn excess conditions and thus interferes with an iron-dependent transcriptional response. To encounter mismetallation and other Mn-dependent problems on a protein level, the cells invest in transcripts of ribosomes, proteases and chaperones. In the case of the ∆*mnx* mutant, the consequences of the disability to export excess Mn from the cytosol manifest in additionally impaired energy metabolism and oxidative stress transcriptomes with a fatal outcome. This study emphasizes the central importance of Mn homeostasis and the transporter Mnx’s role in restoring and holding it.

## Introduction

All organisms rely on an adequate manganese (Mn) supply to maintain the functions of enzymes, such as glycosyl transferases, oxalate oxidase or Mn-dependent superoxide dismutase (SOD) [1,2]. Mn is imported and biologically active in its ionic Mn^2+^ form. Organisms performing oxygenic photosynthesis have a 100-fold higher demand for Mn in comparison to non-photosynthetic organisms since they use Mn (here also bioactive as Mn^3+^ and Mn^4+^ ions) for the oxidation of H_2_O at the oxygen-evolving complex (OEC) [3]. The OEC is a central part of photosystem II (PSII) and hosts the catalytic Mn cluster (Mn_4_CaO_5_), stabilized by PsbO, PsbP and PsbQ in plants and PsbO, PsbU and PsbV in cyanobacteria [4,8]. To ensure proper provision of Mn to the OEC, the model cyanobacterium *Synechocystis* sp. PCC 6803 (hereafter *Synechocystis*) maintains cellular Mn homeostasis. In a light-dependent manner, Mn is imported via outer membrane pores with a low selectivity to the periplasm [4,9]. Here, 75% of the cell’s Mn pool is stored and bound either to the outer membrane or Mn cupin A [4,8]. The remaining 25% are located in the cytoplasm or in the thylakoid system [3,8, 10] and are associated with nucleic acids and small molecule chelates or bound to different metalloproteins [11]. To match the high Mn demand of the OEC, Mn is transported into the cytoplasm by two different Mn import systems. Recently, two members of the unknown protein family 0016 (UPF0016), the hemi manganese exchangers (Hmxs) 1 and 2, were demonstrated to facilitate constitutive Mn uptake via the plasma membrane [12]. Upon limited Mn supply (<1 µM [13]), Hmx1/2 uptake is assisted by the high-affinity ABC-type transporter MntCAB (Mn transporter), which is transcriptionally regulated by the ManSR (manganese sensor/regulator) two-component system [13,15]. If Mn supply is sufficient (>1 µM), Mn^2+^ ions bind to aa residues in the periplasmic loop of the sensor protein ManS. This leads to autophosphorylation of ManS, which phosphorylates the response regulator ManR subsequently. Phosphorylated ManR binds to the promoter of the *mntCAB* operon, blocking its expression. On the contrary, when Mn is scarce, ManS is not activated by phosphorylation, ManR is not phosphorylated and the *mntCAB* operon is expressed, leading to an increased import of Mn [13,14]. Cytoplasmic Mn is either used in the cytoplasm by Mn-requiring enzymes or is further distributed to the thylakoid lumen by the Mn exporter (Mnx) also known as *Synechocystis* photosynthesis-affected mutant 71, another member of the UPF0016 [10,16]. Although it is questionable whether Mn-limited conditions occur in aquatic environments, in lab experiments, very low Mn supply (<0.1 µM) leads to decreased photosynthetic activity since the H_2_O oxidation capacity of the OEC is lowered, and as a consequence, the overall growth rate is reduced [17].

In contrast to Mn limitation, Mn excess has not been studied in detail in cyanobacteria yet. A surplus of Mn results in a decreased chlorophyll *a* content and a reduced photosystem I (PSI) activity at the physiological level and eventually leads to cell death in *Synechocystis* [10,16]. The mechanism of this Mn toxicity is not well understood. Besides the induction of iron (Fe, bioactive as Fe^2+^ ions) limitation, the most plausible mode of action is the mismetallation of enzymes and regulatory proteins, changing or abolishing their activity [8,18]. According to the Irving–Williams series (Mg^2+^<Mn^2+^<Fe^2+^<Co^2+^<Ni^2+^<Cu^2+^>Zn^2+^), different metal ions compete with each other to be bound by aa residues [8,19]. Correct metalation is only favoured due to strictly controlled concentrations of the different metals at the site of metal incorporation during or after protein biosynthesis [20]. The vital importance of controlling the intracellular Mn concentration and subcellular allocation could be demonstrated for the mutant in the thylakoid Mn transporter Mnx [10]. Mnx transports Mn from the cytoplasm into the thylakoid lumen, where the OEC, the highest demand for Mn supply, is located. The knock-out mutant Δ*mnx* displays high light sensitivity and a significantly longer recovery time compared to the WT after photoinhibition, presumably due to the lack of Mn in the thylakoid lumen for enabling the high D1 turnover [10]. It was shown that the ∆*mnx* mutant is highly sensitive towards Mn excess conditions in general and displays a lethal phenotype upon Mn stress as it accumulates Mn intracellularly [10]. Obviously, the subcellular Mn pools need to be carefully maintained at constant levels to ensure proper cell growth, and Mnx plays a critical role in the correct subcellular Mn distribution [10]. However, it is not understood why the cytoplasmic Mn overload in *Synechocystis* is detrimental.

In this study, we aimed at a mechanistic understanding of the Mn excess response in the cyanobacterium *Synechocystis*. To this end, we grew *Synechocystis* cells under standard (9 µM) MnCl_2_ and excess (90 µM) MnCl_2_ conditions and investigated the physiological and transcriptional effects. We studied the WT and the mutant ∆*mnx*, which are defective in the export of Mn from the cytosol. The WT survives 90 µM MnCl_2_ and hence displays an adequate transcriptional response. ∆*mnx* succumbs to 90 µM MnCl_2_ and shows a similar though more pronounced transcriptional response in comparison to the WT. Mn excess induces a transcriptional response similar to Fe limitation with a significantly reduced abundance of transcripts encoding PSI and PSII components, phycobilisomes (PBSs) and Fe uptake systems in both strains. We suggest that mismetallation of the transcriptional regulator, ferric uptake regulator (Fur), is one causative factor. In addition, the ∆*mnx* mutant displays a significant transcriptional reduction of ATPase and carbon metabolism genes in general and shows features of a response towards oxidative stress. Protective mechanisms are not sufficient to compensate for the Mn-dependent mismetallation, energy depletion and reactive oxygen species (ROS) generation in the ∆*mnx* mutant with a fatal outcome.

## Methods

### Cyanobacterial strains and growth conditions

A *Synechocystis* sp. PCC 6803 glucose-tolerant (Japan) strain served as the WT. The ∆*mnx* mutant was generated in a previous study by insertion of a kanamycin resistance cassette into the *mnx* (*sll*0615) ORF [10]. The cells were grown in the BG11 medium, pH 7.5 [21]. Cultivation in Erlenmeyer flasks was performed on a shaker at 100 r.p.m., 30 °C and continuous LED illumination at an intensity of 100 µmol photons m^−2^ s^−1^. For the ∆*mnx* mutant, the medium was supplemented with 50 µg µl^−1^ kanamycin. Before sampling for RNA isolation, the cells were resuspended in a fresh medium and adjusted to an OD_750_ of 0.3, and cell suspensions were split into two flasks per strain. The BG11 medium in one flask was supplemented with a standard concentration of MnCl_2_ (9 µM MnCl_2_). The BG11 medium in the other flask was supplemented with excess MnCl_2_ (90 µM MnCl_2_). All treatments were performed in biological triplicates. After 24-h cultivation, 10 ml samples were taken from each culture and centrifuged in pre-cooled tubes for 10 min at 3000 r.p.m., 4 °C. The cell pellets were snap frozen in liquid N_2_ and stored at −80 °C until further use.

### Drop tests

For the drop test assays, *Synechocystis* cultures were grown as described earlier in the BG11 medium with a standard concentration of 9 µM MnCl_2_ and adjusted to an OD_750_ of 0.2. Dilutions of the cell suspensions 1 : 10, 1 : 100 and 1: 1000 were prepared, and 2 µl of each dilution was dropped onto BG11 agar plates [21] containing different concentrations of MnCl_2_ and/or Fe-NH_4_-citrate. Afterwards, the plates were grown for 5 days at 30 °C under continuous illumination of 100 µmol photons m^−2^ s^−1^.

### Growth performance and pigment measurements

To determine the growth rate of WT and ∆*mnx* under Mn standard conditions and Mn excess conditions, growth curves were generated. For this approach, liquid cultures were grown as described earlier in Erlenmeyer flasks on a shaker. After precultivation, two 50-ml batches of WT and ∆*mnx* mutant with an OD_750_ of 0.2 were filled into growth tubes for the Multi-Cultivator MC-1000-OD (Photon Systems Instruments, Drásov, Czech Republic) and 9 µM or 90 µM MnCl_2_ was added to one tube of WT or ∆*mnx*, respectively. The cultures were grown for 7 days bubbled with filtered ambient air under constant illumination of 100 µmol photons m^−2^ s^−1^ at 30 °C. To estimate the growth performance, OD_750_ was recorded every 20 min.

To calculate the chlorophyll *a*, phycocyanin and carotenoid content, OD_680_ (chlorophyll *a*), OD_625_ (phycocyanin) and OD_490_ (carotenoids), respectively, were measured every 24 h in triplicate. Pigment contents were estimated according to Sigalat and De Kouchkovsky [22].

### RNA-seq analysis

RNA was extracted from cell pellets using the Qiagen RNeasy Plant Mini Kit (Qiagen GmbH, Hilden, Germany) following the manufacturer’s instructions with 10 µl 2-mercaptoethanol per 1 ml RLT buffer. For cell lysis, 500-µl beads with a size of 0.2–0.4 µm were used in a Precellys Evolution cell lyser (Bertin Technologies, Montigny-le-Bretonneux, France) for 4×30 s at 2000 r.p.m. with 15-s pauses in between. RNA was prepared according to the Illumina TruSeq Stranded Total RNA with Illumina Ribo-Zero Plus (Illumina, Hayward, CA, USA). The procedure of rRNA depletion and preparation of the library were conducted as stated in the protocol of the corresponding reference guide from Illumina. The library pool was sequenced using the Illumina NextSeq500 with 76 BP SR (single read) at a high-output flow cell.

All data analyses were performed using R 4.3.0. Differential gene expression analysis was performed with edgeR using the classic test followed by the Benjamini–Hochberg multiple hypothesis correction [23,24]. A principal component analysis (PCA) was performed with transcript per million values using the prcomp function with parameter scale=T and centre=T. Loadings were calculated, and the six highest absolute values for components 1 and 2 were selected and coloured by the highest level Kyoto Encyclopedia of Genes and Genomes (KEGG) [25] map annotation.

### KEGG enrichment

KEGG ontology (KO)-term annotations were retrieved from eggNOG-mapper [26] with default parameters, KEGG Automatic Annotation Server (KAAS) [27] against *Synechocystis* sp. PCC 6803, *Synechocystis elongatus* PCC 7942, *Nostoc* sp. PCC 7120 and *Arabidopsis thaliana*, as well as directly downloaded from KEGG. The CDSs from *Synechocystis* sp. PCC 6803 were used to obtain KO-term annotations from KAAS and eggNOG. Enrichments of significantly differentially abundant transcripts were tested on the ‘KEGG map’ annotation level and the ‘KEGG module’ level for the mutant and control, using Fisher’s exact tests [28]. Upregulated genes were defined as those with a log_2_ fold change >0 with *q*-value <0.05 and downregulated genes as log_2_ fold change <0 and *q*-value <0.05. Enrichments on the PCA eigenvalues were done as described earlier separately for positive and negative values using a cut-off of 0.015 and Fisher’s exact test. Enrichments with a *P*-value ≤0.05 were considered significant.

### Data availability

All code used in this analysis is available on GitLab (https://gitlab.ub.uni-bielefeld.de/computationalbiology/mn-excess-rna-seq; will be made public upon publication). The RNA-seq data set is available at the European Nucleotide Archive with the project ID PRJEB75422. Transcript abundances and statistical data for all genes are provided in Table S1 (available in the online version of this article).

## Results

### Mn excess impairs growth and pigment accumulation of ∆*mnx*

**Fig. 1.**
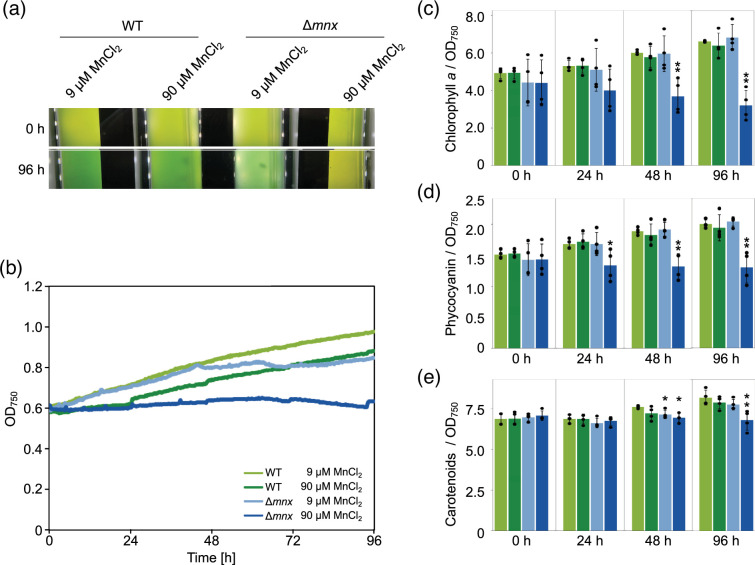
Effects of Mn treatment on growth and pigment content. (**a**) Phenotypic appearance of WT and ∆*mnx* mutant under MnCl_2_ standard (9 µM MnCl_2_) and excess (90 µM MnCl_2_) conditions at different time points in the MC-1000-OD Multi-Cultivator. (**b**) Representative growth curves of WT and ∆*mnx* mutant under different MnCl_2_ regimes. (**c**) Chlorophyll *a* content, (**d**) phycocyanin content and (**e**) carotenoid content in WT and ∆*mnx* cells grown under different MnCl_2_ regimes. Pigment levels were normalized to OD_750_. Significance values in (**c**)–(**e**) were evaluated with the Student’s *t*-test. *P≤0.05. **P≤0.01.

To assess the physiological impact of different Mn regimes on WT and the ∆*mnx* mutant, growth performance and pigment contents were determined. To this end, cells were inoculated into the BG11 medium supplemented with either the standard concentration of MnCl_2_ (9 µM MnCl_2_) or an excess concentration of MnCl_2_ (90 µM MnCl_2_) and grown for 96 h. The concentration of 90 µM MnCl_2_ was chosen, since we found in an earlier study (Brandenburg *et al*. [10]) that 150 µM is immediately deadly to the ∆*mnx* mutant. A 10-fold concentration, that is 90 µM MnCl_2_, was assumed to be critical but not instantly fatal. A pale green phenotype was observed for the ∆*mnx* mutant in comparison to the WT under Mn stress conditions (Fig. 1a). According to their growth curves (Fig. 1b), the WT and ∆*mnx* mutant grew comparably for the first 24 h under standard Mn conditions and similarly reduced after transfer to excess Mn medium. However, the later (after 24 h) growth performance of the ∆*mnx* mutant declined under standard conditions and was fully inhibited under Mn excess conditions. After the initial lag phase, the WT was not affected in growth by the elevated levels of Mn (Fig. 1b). With respect to pigment levels, the ∆*mnx* mutant grown in Mn excess accumulated significantly lower levels of chlorophyll *a* (Fig. 1c) and phycocyanin (Fig. 1d) over time. The carotenoid level was slightly but significantly reduced only after 48 h of growth under Mn excess conditions in the ∆*mnx* mutant (Fig. 1e). On the basis of these results, we selected the timepoint 24 h after the experimental start to study the effects of Mn treatment on the transcriptome of WT and mutant to avoid pleiotropic cytotoxic effects.

### Transcriptional profile of the ∆*mnx* mutant is more strongly affected by Mn excess than the WT

**Fig. 2.**
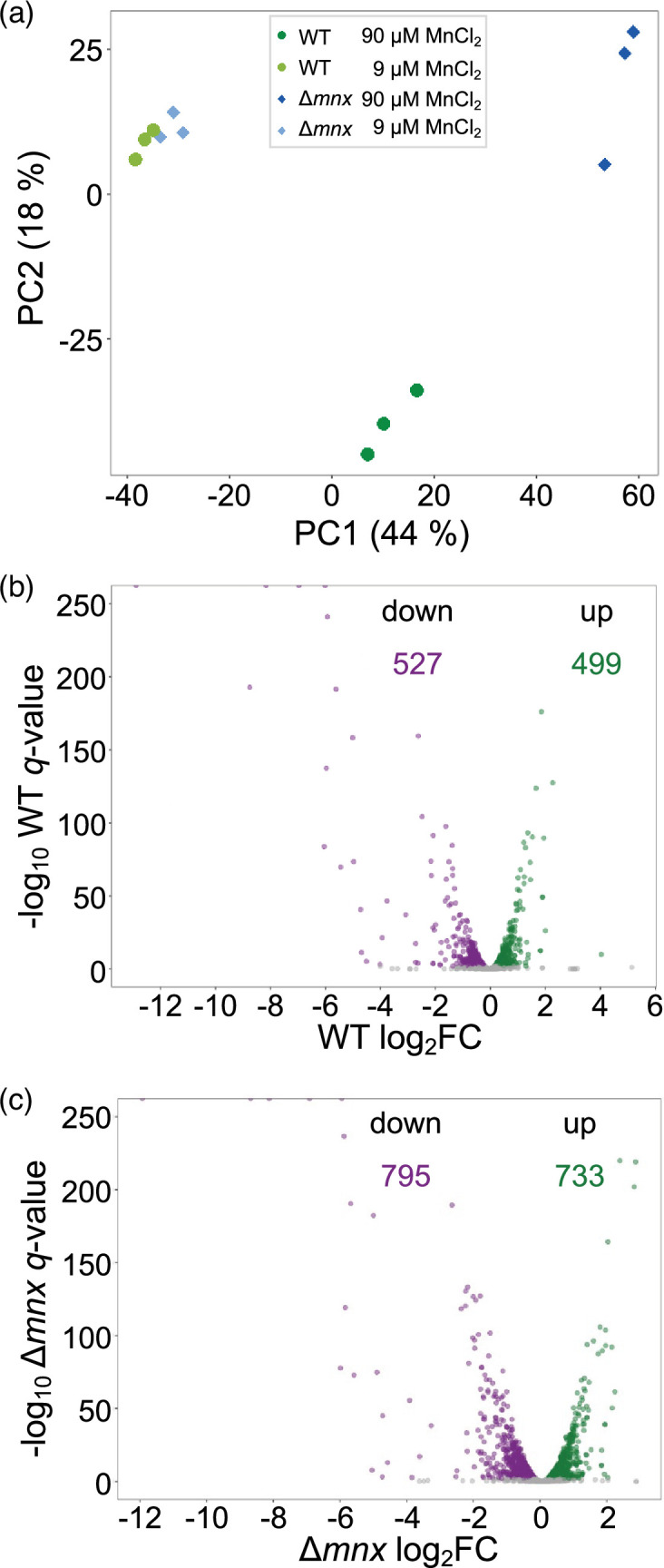
Effect of MnCl_2_ treatment on transcriptomes of WT and ∆*mnx*. (**a**) PCA of transcript abundances in WT and ∆*mnx* cells grown under control and Mn excess (+Mn) conditions. Volcano plots of the global transcriptome responses of WT (**b**) and ∆*mnx* (**c**) towards different MnCl_2_ treatments. Shown are log_2_-fold changes (log_2_FC) of Mn excess (90 µM MnCl_2_) versus Mn control (9 µM MnCl_2_) conditions. Significant changes (*q*<0.01; edgeR, [23]) are plotted in green (up) or violet (down). The number of significantly up- and downregulated genes is given for each genotype.

To assess the effect of excess Mn on the transcriptomes of WT and the ∆*mnx* mutant, transcriptomes of three biological replicates for each strain were analysed after 24 h growth under standard Mn and excess Mn treatment. The PCA of the transcript abundances does not show strong transcriptome differences between the WT and mutant under standard Mn treatment as their samples cluster together (Fig. 2a). The PCA revealed the Mn treatment as the major factor (PC1) contributing to 44% of the variation, while the intracellular Mn allocation likely accounts for the 18% variation in PC2 (Fig. 2a). A larger distance between the Mn excess treated ∆*mnx* to control samples compared to the WT replicates was observed in the first dimension. In addition, WT samples under excess Mn clustered away from all others in the second dimension. This result indicates a quantitative difference in the transcriptional response upon Mn excess in WT and ∆*mnx* cells and a qualitative difference in the response of WT to excess Mn. To gain first insight into the major contributing genes and their functional classification, component loading of the PCA was extracted and enrichment of transcript’s eigenvalues was calculated. The analysis shows enrichments for PC1 for ‘photosynthesis’, ‘carbon fixation’ and ‘ribosomes’ and for PC2 for ‘ribosomes’, ‘photosynthesis, ‘prokaryotic defence’ and ‘rransporters’ (Table S2).

As suggested by the PCA, a comparison of the number of differentially expressed genes (DEGs) upon Mn excess treatment to standard Mn concentrations showed differences between WT and mutant. In the WT, 1026 transcripts were found significantly changed (*q*<0.01, 27.8% of all genes), with 499 genes showing enhanced transcript abundances and 527 genes showing reduced transcript abundances (Fig. 2b) 24 h after Mn excess treatment. Cells of the ∆*mnx* mutant had 1528 significantly (*q*<0.01, 41.4% of all genes) changed transcripts, with 733 genes showing enhanced transcript abundances and 795 genes showing reduced transcript abundances (Fig. 2c). Calculating DEGs between genotypes within a treatment resulted in 25 significantly changed (*q*<0.01, 0.7% of all genes) genes under Mn control conditions (Fig. S1A, Table S3-1) and 862 significantly changed (*q*<0.01, 23.3% of all genes) genes under Mn excess conditions (Fig. S1B, Table S3-2), which agrees with the clustering of samples in the PCA (Fig. 2a). A scatter plot of the analysed transcript abundance in WT and ∆*mnx* showed an apparently linear relationship between the transcriptional response of both WT and ∆*mnx* (Fig. S2). The genes with the strongest transcriptional response had a similar magnitude of response in the mutant and WT, while the genes with a more modest response between fourfold up and down showed a stronger response in the mutant (Fig. S2). The transcripts most impacted by Mn excess in both genotypes are mostly uncharacterized genes located on the extrachromosomal plasmids pSYSM, pSYSA and pSYSX (log_2_-fold change>|1.5|, Table S4). In addition, also *futC* (*sll*1878) and *exbD1* (*sll*1405), which are Fe-responsive genes [29], were severely affected in their transcript abundance (Table S4). A small subset of transcripts falls away from the linear relationship (Fig. S2) and likely represents those loading the PC2 of the PCA (Fig. 2a). These 81 transcripts include 20 with higher abundance in WT but lower in the mutants, such as *rbcL* (*slr*0009) or *petC* (*sll*1316), and 61 with significantly lower abundance in WT but higher in the mutant, such as *hliA* (*ssl*2542) or *ycf64* (*slr*1846) (Table S5). With the exception of a transposase and a gene without annotation, all transcripts are changed less than twofold in WT (Table S5).

These results indicate that the WT and ∆*mnx* display a shared response to Mn stress, but the ∆*mnx* mutant is more affected by Mn excess and the WT shows a small exclusive response.

### Functional transcriptome response largely overlaps between WT and ∆*mnx* mutant

For a detailed and mechanistic understanding of the Mn excess response, we analysed the shared versus the strain-specific transcriptional responses (DEGs with log_2_-fold change≤|1| and *q*≤0.01) of WT and mutant.

**Fig. 3.**
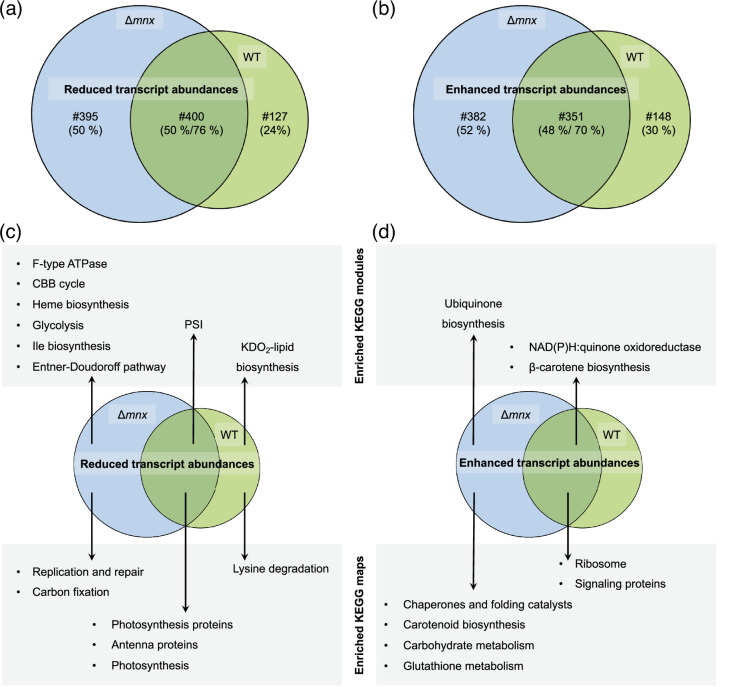
Comparison of the transcriptional response of WT and ∆*mnx* towards Mn excess. The overlap in transcriptional responses upon Mn excess (DEGs with log_2_-fold change≤|1| and *q*≤0.01) is shown with Venn diagrams. The number (#) and percentage (%) of genes, which are shared or specific for ∆*mnx* and the WT, respectively, are given. (**a**) The overlap of genes with significantly reduced transcript abundances in the WT and ∆*mnx.* (**b**) The overlap of genes with significantly enhanced transcript abundances in the WT and ∆*mnx*. (**c**) The overlap of significantly (*P*≤0.05) enriched KEGG modules and maps in significantly reduced transcripts. (**d**) The overlap of significantly (*P*≤0.05) enriched KEGG modules and maps in significantly enhanced transcripts.

The WT and mutant share a response of 751 transcripts (Fig. 3a and b) despite displaying large differences in the growth behaviour (Fig. 1). The 400 shared less abundant transcripts represent 76% of all less abundant WT transcripts but only 50% of the genes with reduced transcript abundance in the ∆*mnx* mutant (Fig. 3a). Among the 351 shared transcripts with enhanced abundances, the overlap was similar with 70% for the WT and 52% for the ∆*mnx* mutant (Fig. 3b).

To gain functional insights, we tested for enrichments of pathways using KEGG map annotations (Table S6) and sub-categories annotated as KEGG modules (Table S7). Twenty-four hours after Mn excess treatment, both the WT and ∆*mnx* mutant showed for the genes with reduced transcript abundances (Fig. 3c) significant enrichment (*P*≤0.05) of the KEGG maps ‘photosynthesis’, ‘photosynthesis proteins’ and ‘antenna proteins’ for their genes. On the more specific KEGG module level, ‘PSI’ was enriched for both strains. The biosynthesis of 3-deoxy-d-manno-octulosonic acid-lipid A, a component of the lipopolysaccharide layer of the outer membrane in the Gram-negative bacteria [30], was the only pathway that was downregulated in the WT according to the KEGG module enrichment as was lysine degradation. The ∆*mnx* mutant solely showed enrichments of several pathways in C metabolism. The KEGG module enrichments (*P*≤0.05) revealed reduced transcripts (Fig. 3c) corresponding to the modules ‘Calvin–Benson–Bassham (CBB) cycle’, ‘glycolysis’, ‘Entner–Doudoroff pathway’ and ‘F-type ATPase’ and corresponding to the maps ‘Ile biosynthesis’, ‘haem biosynthesis’, ‘CO_2_ fixation’ and ‘replication and repair’ only in the ∆*mnx* mutant.

With regard to genes with enhanced transcript abundances upon Mn excess treatment (Fig. 3d), the WT and ∆*mnx* mutant shared enrichment of the categories ‘NAD(P)H : quinone oxidoreductase’, ‘β-carotene biosynthesis’, ‘ribosomes’ and ‘signalling proteins’. While the specific WT response of 148 transcripts did not show any further enrichment, the ∆*mnx* mutant was affected additionally in ‘ubiquinone biosynthesis’, ‘chaperones and folding catalysts’, ‘carotenoid biosynthesis’, ‘carbohydrate metabolism’ and ‘glutathione metabolism’.

### Mn importer MntCAB is reduced in transcript abundance upon Mn excess treatment

**Fig. 4.**
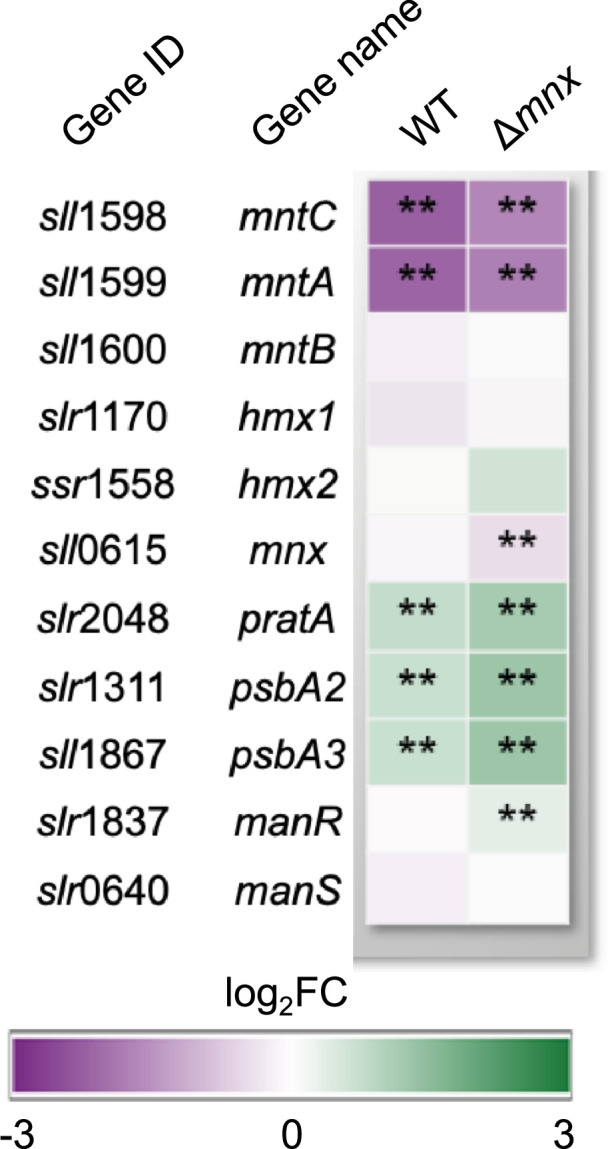
Transcriptional response of genes involved in Mn homeostasis. Transcriptional response upon Mn excess treatment is presented as a heat map of corresponding log_2_-fold change (log_2_FC) for the WT and ∆*mnx* mutant. Statistical differences were evaluated according to Benjamini–Hochberg with *q*≤0.01 (**). Gene loci and names were obtained from Mills *et al*. [59].

We hypothesized that Mn excess affects the expression patterns of genes that encode proteins involved in Mn homeostasis (Fig. 4, [8]). The gene transcripts of the low-Mn inducible high-affinity Mn importer MntCAB [31] were significantly less abundant in both the WT and mutant. The second, constitutive Mn import system Hmx1/2 [12] was not significantly changed in transcript abundances in both strains. In the WT, also the Mnx gene *mnx* was not affected on a transcript level. Transcripts of PratA, which are postulated to function in loading of the pre-D1 protein with Mn, were significantly more abundant with even higher induction in the ∆*mnx* mutant [32]. In accordance, levels of *psbA2* and *psbA3*, encoding D1, were significantly upregulated in both genotypes but even higher in ∆*mnx* upon Mn excess treatment. For the Mn-responsive two-component system ManSR, transcript abundances of ManS were unaffected, while transcript levels of ManR were significantly induced in the mutant only.

### Transcriptional Mn excess response is partially congruent with the response to Fe limitation

**Fig. 5.**
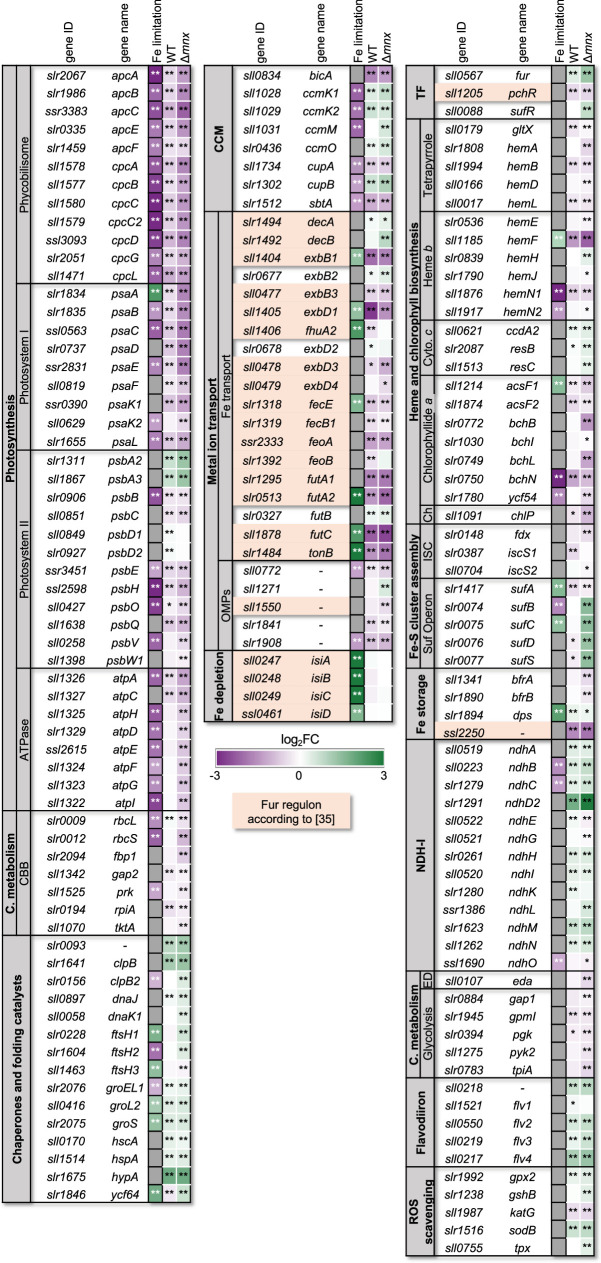
Comparison of Mn excess transcriptional response in WT and ∆*mnx* with Fe limitation response according to Singh *et al*. [29] in WT. Colour coding of the boxes indicates the reduced (violet) or increased (green) transcript abundance of the corresponding gene(s) represented by the mean of the log_2_-fold changes (log_2_FC). Asterisks indicate significant changes with *q*<0.05 (*) and *q*<0.01 (**). Values are given in Table S8. Some data points of the Fe limitation set exceed the colour-coded log_2_-fold change of |3| and are presented in deep violet and green for better comparison. C., carbohydrate; Ch, chlorophyll *a* biosynthesis; Cyto. c, cytochrome *c* biosynthesis.

Since studies on *Escherichia coli* have demonstrated that Mn excess induces Fe limitation [18] and the most affected genes in our work are known to be Fe responsive (Fig. S2), we hypothesized that the Mn excess transcriptomes show features of an Fe limitation response. To test this hypothesis, we compared our transcriptome data to transcriptomic data of Fe limitation in *Synechocystis* for major Fe-responsive pathways (Fig. 5, [29]). Transcript abundances of photosynthesis (PBS, PSI, PSII and ATPase) and CBB cycle corresponding genes were significantly reduced upon Mn treatment as they are in Fe limitation (Fig. 5). Three genes behaved opposite in Mn excess compared to Fe limitation: *psaA* was upregulated in Fe limitation but reduced in Mn excess, and for the D1 encoding genes *psbA2* and *psbA3,* no information was available in Fe limitation but they were upregulated in Mn excess. Fe limitation induces chaperones and proteases [29,33], and in Mn excess, transcripts encoding chaperones and proteases were more abundant (Fig. 5). Other genes from known Fe limitation response pathways reacted under Mn excess conditions in a reverse manner. Transcripts encoding carboxysomal proteins, as part of the carbon-concentrating mechanism, had enhanced transcript levels under Mn excess (Fig. 5). Transcripts of the ATP-binding cassette-type Fe(III) transporter *futABC*, which is the major Fe importer in the plasma membrane, were significantly less abundant under Mn excess conditions, while Fe limitation leads to enhanced transcript levels [34]. Transcript accumulation of other genes encoding Fe importers, such as ferrous iron transport protein B, *feoB*, was significantly reduced under Mn excess in WT only. The energy transmitter genes *tonB* and *exbD1/B1* were significantly reduced in transcript abundance compared to the control as were the genes for outer membrane channel proteins (OMPs). The typical Fe limitation indicative genes, iron stress-induced (isi) genes *isiA* and *isiB*, were not significantly altered in their transcript abundances. The inconsistent pattern led to a non-significant overlap between the transcriptome responses of Mn excess and Fe limitation as tested by hypergeometric distribution calculation (Table S8).

To get a complete picture, we also investigated functional categories associated with the above-mentioned pathways but not specifically mentioned in the iron limitation data set [29]. Studying the expression of transcription factors that are known to be intrinsic regulators of Fe homeostasis in *Synechocystis*, that are Fur, sulphur utilization factor regulator (SufR) and pyochelin regulator (PchR) [35,36], we found for *fur* and *sufR* significantly stronger transcript levels, especially in Δ*mnx*, while *pchR* was reduced (Fig. 5). Connected to the light reactions of photosynthesis, we detected mostly reduced transcript levels of genes involved in haem and chlorophyll biosynthesis with the exception of cytochrome *c* biogenesis in Mn excess and mixed pattern in significantly changed transcripts of the Fe limitation data (Fig. 5). Fe–S cluster biogenesis is an exception to shared WT and mutant excess Mn responses. It is majorly operated by the sulphur utilization factor (Suf) system in *Synechocystis* and was only enhanced on the transcript level of the *sufBCDS* operon in the Δ*mnx* mutant again with mixed responses observed in Fe limitation (Fig. 5). With regard to Fe storage, the transcriptional response to Mn excess is varied. The two bacterioferritin (bfr) family protein genes *bfrA* and *bfrB* [37] were both reduced on a transcript level in Δ*mnx*. The bfr-associated ferredoxin encoding gene *ssl*2250 [35] was strongly downregulated in both genotypes, while unaffected under Fe limitation. The gene *slr*1894, encoding an Fe storage protein of the Dps family [38], was enhanced in transcript abundance under both Fe limitation and Mn excess. The transcripts encoding subunits of NAD(P)H:chinon-oxidoreductase (NDH-1) complexes, which are central components of respiration, cyclic electron flow and intracellular CO_2_ accumulation [39], were upregulated in Mn excess with the sole exception of *ndhO* but opposite in Fe limitation for the two significant data points. Glycolytic enzymes were reduced in transcripts upon Mn excess treatment with no significant changes in Fe limitation. With regard to the prevention of ROS formation, we observed enhanced transcript abundances of the flavodiiron proteins Flv2, Flv3 and Flv4, which serve as alternative electron sinks at the acceptor side of PSI [40] with no significant changes in Fe limitation. Furthermore, ROS scavenging by SOD and glutathione appeared stronger on a transcript level and again no significant changes in Fe limitation were determined.

Taken together, the comparison of the Mn excess transcriptome with Fe limitation transcriptome showed a partial overlap, however, with contrasting patterns to some extent, while the Fe-limitation signature genes *isiABCD* were unaffected under Mn excess conditions. Effects were stronger in the Δ*mnx* mutant.

### Fe surplus does not rescue the Mn excess phenotype in ∆*mnx*

**Fig. 6.**
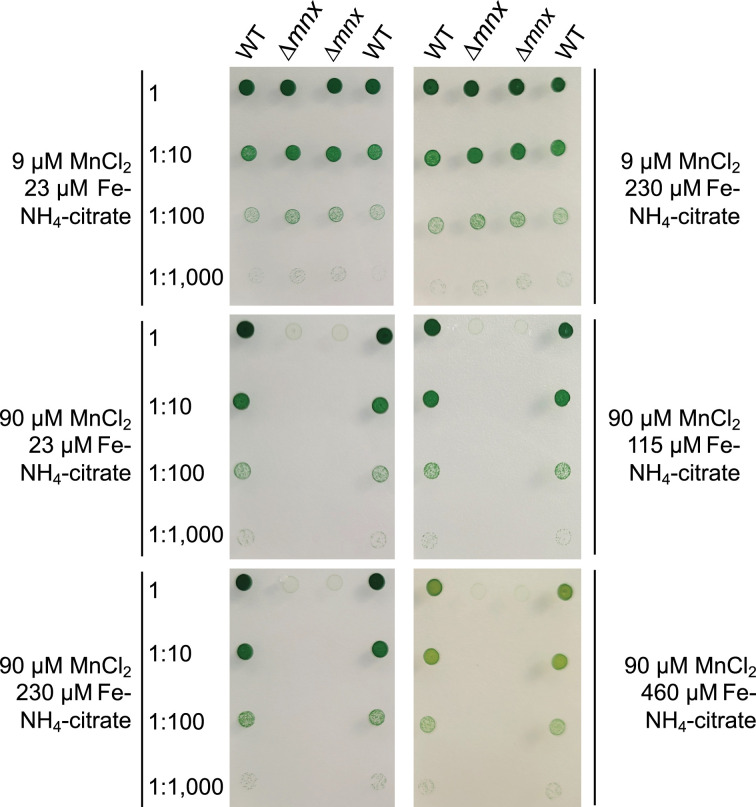
Growth of WT and ∆mnx mutant under different Mn/Fe treatments. Growth of different dilutions (1, 1:10, 1:100, 1 :1000) was investigated on the BG11 medium supplemented with standard Mn/Fe concentrations (9 µM MnCl_2_; 23 µM Fe–NH_4_–citrate), standard Mn (9 µM MnCl_2_) and surplus Fe (230 µM Fe–NH_4_–citrate) or on excess Mn (90 µM MnCl_2_) and increasing Fe concentrations (23–460 µM Fe–NH_4_–citrate). Plates were photographed after 5-d incubation under continuous illumination with 100 µmol photons m^−2^ s^−1^ at 30 °C.

Based on the results from the transcriptome data, which were in parts similar to a Fe limitation response, we hypothesized that additional Fe supplementation shall rescue the growth of *∆mnx* upon Mn excess. Accordingly, we performed growth tests (Fig. 6) on BG11 plates with standard (9 µM MnCl_2_) or excess (90 µM MnCl_2_) Mn concentrations and increasing Fe–NH_4_–citrate concentrations (standard 23 µM Fe–NH_4_–citrate to 20-fold, that is 460 µM Fe–NH_4_–citrate). While a growth difference between the WT and *∆mnx* mutant at a standard Mn concentration was not obvious, the lethal phenotype of the *∆mnx mutant* under Mn excess conditions could not be compensated by extra supplementation with Fe up to a 20-fold increased concentration (Fig. 6).

## Discussion

Mn toxicity is a poorly understood process. It is clear that the cellular Mn load needs to be controlled within narrow borders. For single-cell organisms, such as *Vibrio cholerae*, *E. coli* or *Synechocystis*, it was observed that already a two- to threefold increased Mn loading was lethal when the main Mn export system was knocked out [10,16, 18, 41, 42]. So far, only for *E. coli,* a detailed study of the Mn excess response has been performed [18]. In this organism, Mn excess leads to Fe deficiency. As a consequence, Fe–S cluster assembly and haem biogenesis are impaired. This leads to a disruption of Fe-dependent electron transport chains and a block of the tricarboxylic acid cycle, causing an ATP crisis, which affects vital cellular processes. Additionally, the production of ROS induces DNA damage and affects protein stability [18]. In contrast to heterotrophic bacteria like *E. coli*, cyanobacteria have an at least 100-fold higher demand for Mn, since they utilize the micronutrient as the inorganic catalyst of light-driven water oxidation [4]. Thus, it is possible that Mn homeostasis and its regulatory network work differently.

### Excess Mn treatment does not induce Fe limitation in *Synechocystis*

**Fig. 7.**
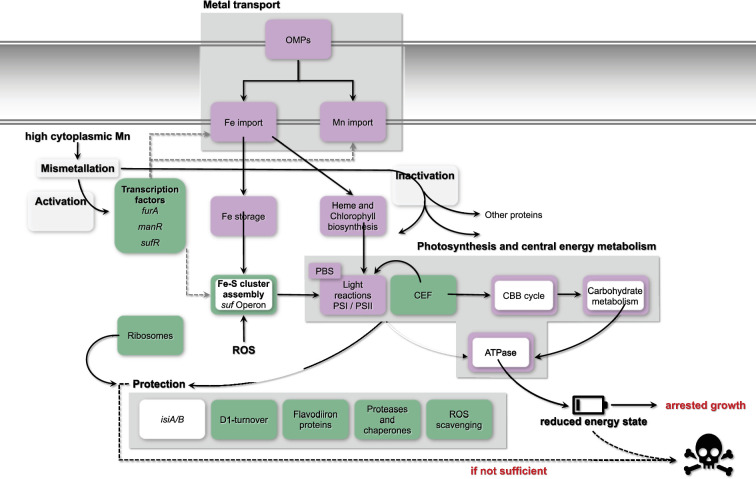
Model for an Mn excess response in *Synechocystis*. Presented are the effects of an Mn excess treatment on the global transcriptome and physiology of *Synechocystis* WT and ∆*mnx* mutant, which are defective in Mn efflux. The tile colour indicates whether genes of the functional category were enhanced (green), reduced (violet) or unchanged (white) in transcript abundance 24 h after the application of Mn excess stress in both, the WT and ∆*mnx* mutant. Responses that are exclusive to the Δ*mnx* mutant are displayed by a colour-framed tile. The response of specific transcripts is given in Fig. 5 and Table S1. Transcription factors related to Fe (Fur), Mn import (ManR) and Fe–S cluster assembly (SufR) are transcriptionally upregulated. Depending on the cytoplasmic Mn status, Fur likely gets activated by mismetallation with Mn instead of Fe and conveys a transcriptional response that is partially overlapping with an Fe acclimation response in *Synechocystis*. Cellular entrance of both Mn and Fe gets reduced due to the downregulation of Fe and Mn import systems as well as OMPs. Importantly, transcripts of the Fe-deficiency responsive operon *isiABCD* are not altered, indicating that cells are not suffering from Fe limitation. Fe-dependent haem and chlorophyll biosynthesis is downregulated on a transcript level, overall leading to a lower abundance of photosynthesis, that is light harvesting via PBSs, light reactions and carbon reactions (CBB cycle) and carbohydrate metabolism-related gene transcripts. As an additional negative effect, typically Fe-containing proteins involved in these and further processes are likely mismetallated with Mn and thus inactivated. Together with downregulation of ATPase corresponding gene transcripts, cellular energy levels become depleted, with arrested cell growth as an outcome. To cope with the detrimental effects of Mn excess, several protection mechanisms (D1 turnover, flavodiiron proteins, ROS scavenging, proteases and chaperons) are enhanced on a transcript level to prevent cell death. In the case of the ∆*mnx* mutant, the Mnx is not operative. The mutant is not able to adjust cytoplasmic Mn homeostasis. Consistently exclusive to ∆*mnx* mutant cells is the enhanced expression of the *sufBCDS* operon, involved in Fe–S cluster assembly, which indicates ROS stress in those cells upon Mn stress treatment. The protection mechanisms to deal with ROS and also mismetallation effects are insufficient and lead to cell death. CEF, cyclic electron flow.

In general, the WT transcriptomes were affected by the Mn treatment after 24 h, though to a rather mild extent in comparison to the Δ*mnx* mutant. In accordance with Mn stress-induced Fe depletion observed in *E. coli* [18], we detected features of a typical Fe limitation response in the cyanobacterium *Synechocystis*. This response included reduced transcript abundances of genes involved in chlorophyll and haem biosyntheses, photosynthesis covering both light and carbon reactions, carbon catabolism and respiration (Figs 5 and 7). However, the overlap between the Mn excess transcriptomes and a representative Fe limitation transcriptome [29] was not significant. We also compared our data with further Fe limitation transcriptomes [35,43, 44], coming to the same result (Table S8). Not being Fe limited is additionally supported by the unaffected levels of *isiABCD* (Fig. 5). Induction of this operon is considered as a hallmark for Fe limitation in cyanobacteria [45]. Thus, we suggest that the partially congruent response is not caused by the cellular Fe status but due to the cross-regulation of metal-responsive transcriptional regulators.

### Mismetallation of the transcriptional regulator Fur likely triggers parts of transcriptional response towards Mn excess

A reasonable explanation for the partially Fe-dependent transcriptional response is possible crosstalk of transcriptional regulators. Fur is generally considered as a key regulator in bacterial Fe homeostasis [46]. According to our data, the transcript abundance of *fur* is significantly increased in both strains (Figs5  7), which was also described by Sharon *et al.* [43] upon Fe limitation in *Synechocystis*. Many targets of the Fur regulon are affected during Mn excess conditions (Table S9), however in an unexpected way. The Fur-regulated Fe importers FhuA, FutABC, FeoAB, TonB/ExbBD and FecBCDE are under Fe limitation typically upregulated [35] but under Mn stress downregulated (Fig. 5). The transcriptome data suggest that Fur itself commits besides an Fe- and also an Mn-dependent response. In the case of Fe binding by Fur, the binding of one Fe^2+^-ion per monomer induces the dimerization of Fur and thereby enables the binding of the transcription factor to Fur boxes. Mostly, Fur acts as a repressor, for example, represses the expression of Fe importers upon Fe sufficiency, but can also act as an indirect activator via the repression of regulatory antisense RNAs [46]. However, Fur was demonstrated to bind not only Fe but also Mn in *Bacillus subtilis*, that is mismetallation of Fur, leading to inappropriate repression of Fe-uptake proteins [47]. This is in line with our observation of transcriptional reduction of Fe import proteins (Figs5  7) and at least partially explains the transcriptional response being similar to an Fe acclimation response. It is furthermore conceivable that Mn binding not only changes the activity of the transcriptional regulator with regard to repression/activation but also enables control of regulons different from those typically known for Fe–Fur. Mismetallation appears in the case of Fur to be rather advantageous and not a collateral damage.

With regard to Mn-dependent transcriptional regulation, heterotrophic bacteria mainly use two different mechanisms: Mn-binding transcription factors, i.e. MntR, and Mn-binding riboswitches, i.e. the *yybP-ykoY* riboswitch (reviewed in [11]). In contrast, little is known about cyanobacteria. So far, only the ManSR two-component system has been identified as being involved in the regulation of Mn homeostasis in cyanobacteria [13,15,43]. In agreement with the high external Mn concentration in our study, the expression of the response regulator ManR was increased (Fig. 4) and the response regulator likely phosphorylated by ManS. As a result, the binding of phosphorylated ManR repressed transcription of its primary target, the *mntCAB* operon, limiting further Mn uptake via this system (Figs4  7).

Consistently with studies on Fe and/or Mn limitation [43], we observed cross-regulation of genes playing central roles in Mn and Fe homeostases. We suggest a likely interconnection of Fur and ManR or yet unknown Mn-dependent regulators integrates signals and orchestrates an appropriate transcriptional response enabling to finally deal with the stress situation. Future studies will reveal the function of transcriptional regulators in maintaining Mn homeostasis.

### Diminishment of Mn uptake comes along with reduced Fe uptake

According to Kaur *et al.* [18], Mn stress in *E. coli* induces down-regulation on the transcriptional level of Fe import and biosynthesis genes for the Fe siderophore enterobactin, leading to an Fe-limitation phenotype. Upon extra addition of Fe to the medium, the transcript abundances of Fe import systems were raised, and thus, it was possible to rescue the Mn stress phenotype in the *E. coli* mutant in the Mn efflux pump MntP [18]. We observed a likewise downregulation of Fe import systems but were not able to compensate Mn toxicity in the *Synechocystis Δmnx* mutant by supplementation with extra Fe (Fig. 6). We suggest that this observation is due to the occurrence of different uptake mechanisms in both organisms.

*E. coli* is an Fe-centric bacterium that does not rely on Mn except for ROS scavenging. Hence, for Fe uptake, several transporters exist, such as FecABCDE, FepBCDG, FhuA and FeoAB [48,50], while Mn import is facilitated by the highly specific Mn importer MntH [51]. Upon Mn limitation and oxidative stress, MntH expression is induced to facilitate Mn influx [52]. In contrast, cyanobacteria, such as *Synechocystis*, are dependent on both, abundant Fe and Mn supplies. Efficient Mn uptake to ensure Mn-dependent photosynthetic water-splitting activity [4] is realized by the use and interplay of the inducible high-affinity MntCAB and the constitutive Hmx1/2 [12] system at the plasma membrane and Mnx [10,16] at the thylakoid membrane [53]. Also, the mainly Fe-transporting FutABC system likely supports Mn import, however in a low-affinity manner [8,12, 43]. The entrance via the outer membrane into the periplasm is suggested to be shared between Fe, Mn and other metals. According to the transcriptional profiles, the overaccumulation of Mn leads to decreased transcription of genes encoding the Mn import systems MntCAB (Fig. 4) and FutABC (Fig. 5). As a result, further efficient Mn uptake is likely reduced or stopped to prevent the cell from damage due to the accumulation of intracellular Mn (Fig. 7). According to its suggested house-keeping function [12], transcript levels of the Hmx1/2 Mn transporter remained unaltered. The Mnx was not affected in the WT on a transcriptional level (Fig. 4). Possibly, to fully abolish Mn uptake, also components for the uptake via the outer membrane, *tonB*, *exbB1* and *exbD1* [35], were significantly lowered in transcript levels. The transcriptional repression is again explainable with mismetallated Fur, since it acts as a transcriptional regulator of those genes. As a consequence, Fe uptake using the same outer membrane passage was hindered, too. Interestingly, Sharon and coworkers [43] already postulated a common transcriptional response of certain Fe and Mn transporters under Fe- and/or Mn-limiting conditions. A shared path of Fe and Mn was furthermore supported by the finding that the addition of surplus Fe did not rescue the Δ*mnx* mutant from death under Mn excess conditions (Fig. 6). Furthermore, this result also fosters the notion that Fe limitation was not the reason for cell death of the Δ*mnx* mutant upon excess Mn treatment. The Fe uptake systems are downregulated but not fully repressed on a transcriptional level. Thus, extra Fe supply would have enabled enhanced Fe uptake by the cells and compensated a possible limitation phenotype. However, the treatment did not rescue the Δ*mnx* mutant and again supports together with the unaffected expression of *isiABCD* (Fig. 5) that Fe limitation was not causing the fatal outcome of Mn excess treatment in the Δ*mnx* mutant.

### Detrimental effects of Mn excess are fought on several levels

The physiological response of the WT with stable growth and pigmentation (Fig. 1) indicated that the WT was able to handle the extra Mn and took measures to restore Mn homeostasis after the application of excess Mn and/or to function in excess Mn. Likely, the Mnx enabled efficient efflux of Mn surplus and recovery of at least adequate cellular Mn pools that, if combined with the observed transcriptional changes, allowed unaffected growth.

Mismetallation is a common event when metal homeostasis is disturbed [54]. Prime targets for mismetallation are Fe-containing proteins. PSI, which is also transcriptionally downregulated (Figs3c  5), is the major target of Mn excess within the PS apparatus of plants, such as *A. thaliana* [55], and possibly also in *Synechocystis*. As a consequence, photosynthetic electron transfer is impaired and entails a highly delicate challenge for oxygenic photosynthetic organisms. To prevent or deal with the formation of ROS, the transcriptomes inform about possible strategies *Synechocystis* employs (Fig. 7): (i) transcript levels of Flv proteins Flv2/3/4 are enhanced (Fig. 5) to serve as an alternative electron sink at PSI [40]; (ii) transcript abundances of ROS scavenger proteins (SodB, GPX cycle, Fig. 5) are upregulated; (iii) *psbA* is upregulated in expression for accelerated PSII turnover (Fig. 4) and (iv) to fight issues on a protein level that come with mismetallation, such as misfolding or inhibited enzyme activity, protease and chaperones (e.g., FtsH1/2, GroEL1/L2/S, ClpB/B2, DnaJ/K1) are more efficiently transcribed (Fig. 5), as also indicated by KEGG map enrichment for chaperons and folding catalysts in the *∆mnx* mutant (Fig. 3d, Table S6). Overall, it was obvious that after Mn excess treatment, transcript levels of proteins with rather stable pools, such as photosynthesis or antenna proteins, were downregulated, while in contrast transcripts related to ribosomes were enhanced (Fig. 3c and d). This finding indicates that cells rather invest in ribosomes (Figs3  7) likely to foster biosynthesis of proteins with higher turnover due to being either damaged by ROS (e.g. PsbA), mismetallated or misfolded.

### One causative factor of Mn intoxication in Δ*mnx* is reduced energy metabolism

In contrast to the WT, the Δ*mnx* mutant was strongly negatively impacted by Mn in growth and pigmentation (Fig. 1). To investigate the nature/reason of Mn toxicity, the Δ*mnx* mutant is a reasonable study object since Mn efflux is hindered in this strain due to the deletion of Mnx [10,16]. An approximately threefold enhanced intracellular Mn load was demonstrated [10,16]. Basically, the Δ*mnx* mutant mounts an adequate response on a transcriptional level that corresponds with the WT response. Alterations that are exclusive to the mutant may indicate critical effects of Mn toxicity.

Besides transporters, Δ*mnx*-exclusive large changes on a transcriptional level after Mn excess application were detected for the categories ‘photosynthesis’ and ‘central carbon metabolism’ (Figs3, 5  7). Photosynthesis as the basis of the energy metabolism for oxygenic photosynthetic organisms is highly Fe dependent: chlorophyll biosynthesis is directly linked to haem biosynthesis and PSI together with the cytochrome*–b_6_f* complex requires a total of 12 Fe atoms, which are mainly used as cofactors or as Fe–S clusters [34]. Genes encoding proteins of the light reactions (PSI, PSII, PBS, pigment biosynthesis and cytochrome *b_6_f* complex) were reduced in their transcript abundances in general under Mn excess conditions (Fig. 5), as also indicated by the component loading of PC1 (Table S2) and KEGG map enrichment (Fig. 3c, Table S6). Reduction in pigment accumulation, that is chlorophyll, phycocyanin and also carotenoids, was clearly detectable also on a physiological level in the ∆*mnx* mutant after adding excess Mn (Fig. 1c, d and e) and is considered as a typical sign of Mn intoxication [56], as the reduction in PSI is [55]. Besides photosynthesis, our results furthermore showed that transcripts corresponding to the ATPase were significantly reduced in abundance (Figs3c  5, Table S8). We postulate that light harvesting and photosynthetic electron transport become consequently impaired under Mn excess conditions, finally manifesting in reduced generation of ATP and reduction equivalents. The depletion in reduction equivalents goes hand in hand with impaired CO_2_ fixation due to reduced transcript levels of ribulose-1,5-bisphosphate carboxylase/oxygenase (Rubisco) subunits and enzymes of the CBB cycle (Figs3c  5). Switching to heterotrophic lifestyle is not assistant, since also genes encoding glycolytic enzymes are together with ATPase downregulated (Figs3c  5). As indicated by higher transcript levels of NDH-1 (Fig. 5), cells enhance cyclic electron transfer to match the reduced NADH+H^+^ consumption due to reduced CO_2_-fixation capacity via Rubisco and the CBB cycle but current cellular ATP need. In summary, our results from the RNA-seq analysis indicate a significant reduction of ATP generation and energy metabolism in general (Fig. 7).

### Oxidative stress is concurrently causative for Mn intoxication in Δ*mnx*

Another obvious differential feature of the transcriptional response in the ∆*mnx* mutant upon Mn treatment is the induction of the *sufBCDS* operon. The operon encodes for proteins of the Suf system, which is central to the biogenesis of Fe–S cluster proteins in cyanobacteria and other bacteria (reviewed in Pérard and Ollagnier de Choudens [57]). Expression of the operon is under the control of the transcription regulator SufR, which binds one [4Fe–4S] cluster per subunit, forms homodimers as holoproteins and acts as a repressor upon binding. The Fe–S cluster functions as a sensor for Fe availability and cellular redox status [58]. Oxidative stress causes damage to the SufR Fe–S clusters. As a result, the binding affinity of SufR to the *sufBCDS*-promoter region is reduced and the expression of the *sufBCDS* operon is not any longer repressed [36,58]. Accordingly, we interpret the enhanced expression of the operon as an indication for sensing of enhanced oxidative stress in ∆*mnx* as also noted by enhanced expression of ROS scavenging enzymes (Figs5  7).

The enhanced ROS generation is possibly a consequence of the cytoplasmic Mn overload and concurrent mismetallation events, which impair photosynthetic electron transfer. Ample chaperones and folding catalysts are up in ∆*mnx* (Figs3d  5), indicating tightened protein quality issues. However, while the WT was able to adjust cytosolic Mn homeostasis by Mnx-catalysed Mn efflux, the mutant just tipped over the edge of tolerable cytoplasmic Mn concentration, and the sum of protective mechanisms is not sufficient to alleviate Mn toxicity. The central importance of Mnx in maintaining cytoplasmic Mn homeostasis is highlighted by the observation that *Synechocystis* WT thrives even on 400 µM MnCl_2_ tested [10]. Without Mnx, the ultimate outcome of high cytoplasmic Mn load due to impaired Mn efflux is cell death (Fig. 7).

### Conclusions

The cyanobacterium *Synechocystis* tolerates treatments with high MnCl_2_ concentrations without negative effects on its performance. Respective transcriptional profiles indicate mismetallation of the canonical Fe-regulated transcription regulator Fur, enabling cross-regulation of Mn- and Fe-responsive genes. Investment into ribosomes likely enables compensation of Mn-dependent mismetallation and protein damage. In the case of impaired Mn efflux by Mnx, cytoplasmic Mn accumulation acts toxic by shutting down central parts in energy metabolism covering both photosynthesis and respiration. Emerging ROS generation cannot be sufficiently compensated by protective measures making the effects of Mn intoxication in the ∆*mnx* mutant fatal. Our analyses thus reveal (i) that Mnx is not involved in sensing and transmission of cellular Mn status, but (ii) Mnx is of absolute importance in balancing cytoplasmic Mn homeostasis.

## supplementary material

10.1099/mic.0.001515 Uncited Supplementary Material 1.

10.1099/mic.0.001515 Uncited Supplementary Material 2.

## Abbreviations

bfr: bacterioferritin
CBB: Calvin–Benson–Bassham
DEGs: differentially expressed genes
Fur: ferric uptake regulator
Hmx: hemi manganese exchanger
KEGG: Kyoto encyclopedia of genes and genomes
KO: KEGG ontology
ManR: manganese regulator
ManS: manganese sensor
ManSR: manganese sensor/regulator
Mn: manganese
Mnx: Mn exporter
NDH-1: NAD(P)H : chinon-oxidoreductase
OEC: oxygen-evolving complex
PBS: phycobilisome
PchR: pyochelin regulator
PSI: photosystem I
PSII: photosystem II
ROS: reactive oxygen species
Rubisco: ribulose-1,5-bisphosphate carboxylase/oxygenase
Suf: sulphur utilization factor
SufR: sulphur utilization factor regulator
UPF0016: unknown protein family 0016

## References

[1] Chandler LE, Bartsevich VV, Pakrasi HB (2003). Regulation of manganese uptake in *Synechocystis* 6803 by RfrA, a member of a novel family of proteins containing a repeated five-residues domain. Biochemistry.

[2] Sevilla F, López-Gorgé J, Gómez M, del Río LA (1980). Manganese superoxide dismutase from a higher plant. Planta.

[3] Stengel A, Gügel IL, Hilger D, Rengstl B, Jung H (2012). Initial steps of photosystem II de novo assembly and preloading with manganese take place in biogenesis centers in *Synechocystis*. Plant Cell.

[4] Keren N, Kidd MJ, Penner-Hahn JE, Pakrasi HB (2002). A light-dependent mechanism for massive accumulation of manganese in the photosynthetic bacterium *Synechocystis* sp. PCC 6803. Biochemistry.

[5] De Las Rivas J, Heredia P, Roman A (2007). Oxygen-evolving extrinsic proteins (PsbO,P,Q,R): bioinformatic and functional analysis. Biochimica et Biophysica Acta (BBA) - Bioenergetics.

[6] Zhang B, Zhang C, Liu C, Jing Y, Wang Y (2018). Inner envelope chloroplast manganese transporter 1 supports manganese homeostasis and phototrophic growth in *Arabidopsis*. Mol Plant.

[7] Xiao Y, Huang G, You X, Zhu Q, Wang W (2021). Structural insights into cyanobacterial photosystem II intermediates associated with Psb28 and Tsl0063. Nat Plants.

[8] Eisenhut M (2020). Manganese homeostasis in cyanobacteria. *Plants*.

[9] Duy D, Soll J, Philippar K (2007). Solute channels of the outer membrane: from bacteria to chloroplasts. Biol Chem.

[10] Brandenburg F, Schoffman H, Kurz S, Krämer U, Keren N (2017). The *Synechocystis* manganese exporter Mnx is essential for manganese homeostasis in cyanobacteria. Plant Physiol.

[11] Waters LS (2020). Bacterial manganese sensing and homeostasis. Curr Opin Chem Biol.

[12] Reis M, Brandenburg F, Knopp M, Flachbart S, Bräutigam A (2023). Hemi manganese exchangers 1 and 2 enable manganese import at the plasma membrane in cyanobacteria. Plant Biol.

[13] Yamaguchi K, Suzuki I, Yamamoto H, Lyukevich A, Bodrova I (2002). A two-component Mn2+-sensing system negatively regulates expression of the mntCAB operon in *Synechocystis*. Plant Cell.

[14] Zorina A, Sinetova MA, Kupriyanova EV, Mironov KS, Molkova I (2016). *Synechocystis* mutants defective in manganese uptake regulatory system, ManSR, are hypersensitive to strong light. *Photosynth Res*.

[15] Ogawa T, Bao DH, Katoh H, Shibata M, Pakrasi HB (2002). A two-component signal transduction pathway regulates manganese homeostasis in *Synechocystis* 6803, a photosynthetic organism. J Biol Chem.

[16] Gandini C, Schmidt SB, Husted S, Schneider A, Leister D (2017). The transporter SynPAM71 is located in the plasma membrane and thylakoids, and mediates manganese tolerance in *Synechocystis* PCC6803. New Phytol.

[17] Salomon E, Keren N (2011). Manganese limitation induces changes in the activity and in the organization of photosynthetic complexes in the cyanobacterium *Synechocystis* sp. strain PCC 6803. Plant Physiol.

[18] Kaur G, Kumar V, Arora A, Tomar A, Ashish (2017). Affected energy metabolism under manganese stress governs cellular toxicity. Sci Rep.

[19] Irving H, Williams RJP (1948). Order of Stability of metal complexes. Nature.

[20] Foster AW, Young TR, Chivers PT, Robinson NJ (2022). Protein metalation in biology. Curr Opin Chem Biol.

[21] Stanier RY, Deruelles J, Rippka R, Herdman M, Waterbury JB (1979). Generic assignments, strain histories and properties of pure cultures of cyanobacteria. J Gen Microbiol.

[22] Sigalat C, De Kouchkovsky Y (1975). Fractionnement et caracterisation de l’appareil photosynthetique de l’algue bleue unicellulaire *Anacystis nidulans*. I. obtention de fractions membranaires par “lyse osmotique” et analyse pigmentaire. Physiol Veg.

[23] Robinson MD, McCarthy DJ, Smyth GK (2010). edgeR: a bioconductor package for differential expression analysis of digital gene expression data. Bioinformatics.

[24] Benjamini Y, Hochberg Y (1995). Controlling the false discovery rate: a practical and powerful approach to multiple testing. J R Stat Soc Series B Stat Methodol.

[25] Kanehisa M, Furumichi M, Tanabe M, Sato Y, Morishima K (2017). KEGG: new perspectives on genomes, pathways, diseases and drugs. Nucleic Acids Res.

[26] Cantalapiedra CP, Hernández-Plaza A, Letunic I, Bork P, Huerta-Cepas J (2021). eggNOG-mapper v2: functional annotation, orthology assignments, and domain prediction at the metagenomic scale. Mol Biol Evol.

[27] Moriya Y, Itoh M, Okuda S, Yoshizawa AC, Kanehisa M (2007). KAAS: an automatic genome annotation and pathway reconstruction server. Nucleic Acids Res.

[28] Fisher RA (1922). On the interpretation of χ2 from contingency tables, and the calculation of P. J R Stat Soc.

[29] Singh AK, McIntyre LM, Sherman LA (2003). Microarray analysis of the genome-wide response to iron deficiency and iron reconstitution in the cyanobacterium *Synechocystis* sp. PCC 6803. Plant Physiol.

[30] Opiyo SO, Pardy RL, Moriyama H, Moriyama EN (2010). Evolution of the Kdo2-lipid a biosynthesis in bacteria. BMC Evol Biol.

[31] Bartsevich VV, Pakrasi HB (1995). Molecular identification of an ABC transporter complex for manganese: analysis of a cyanobacterial mutant strain impaired in the photosynthetic oxygen evolution process. EMBO J.

[32] Nickelsen J, Rengstl B (2013). Photosystem II assembly: from cyanobacteria to plants. Annu Rev Plant Biol.

[33] Hernández-Prieto MA, Schön V, Georg J, Barreira L, Varela J (2012). Iron deprivation in *Synechocystis*: inference of pathways, non-coding RNAs, and regulatory elements from comprehensive expression profiling. *G3 (Bethesda*).

[34] Kroh GE, Pilon M (2020). Regulation of iron homeostasis and use in chloroplasts. Int J Mol Sci.

[35] Riediger M, Hernández-Prieto MA, Song K, Hess WR, Futschik ME (2021). Genome-wide identification and characterization of Fur-binding sites in the cyanobacteria *Synechocystis* sp. PCC 6803 and PCC 6714. DNA Res.

[36] Wang T, Shen G, Balasubramanian R, McIntosh L, Bryant DA (2004). The sufR gene (sll0088 in Synechocystis sp. strain PCC 6803) functions as a repressor of the sufBCDS operon in iron-sulfur cluster biogenesis in cyanobacteria. J Bacteriol.

[37] Keren N, Aurora R, Pakrasi HB (2004). Critical roles of bacterioferritins in iron storage and proliferation of cyanobacteria. Plant Physiol.

[38] Shcolnick S, Shaked Y, Keren N (2007). A role for mrgA, A DPS family protein, in the internal transport of Fe in the cyanobacterium *Synechocystis* sp. PCC6803. *Biochimica et Biophysica Acta (BBA) - Bioenergetics*.

[39] Hualing M (2022). Cyanobacterial NDH-1 complexes. Front Microbiol.

[40] Mustila H, Muth-Pawlak D, Aro EM, Allahverdiyeva Y (2021). Global proteomic response of unicellular cyanobacterium *Synechocystis* sp. PCC 6803 to fluctuating light upon CO_2_ step-down. Physiol Plant.

[41] Waters LS, Sandoval M, Storz G (2011). The *Escherichia coli* MntR miniregulon includes genes encoding a small protein and an efflux pump required for manganese homeostasis. J Bacteriol.

[42] Fisher CR, Wyckoff EE, Peng ED, Payne SM (2016). Identification and characterization of a putative manganese export protein in *Vibrio cholerae*. J Bacteriol.

[43] Sharon S, Salomon E, Kranzler C, Lis H, Lehmann R (2014). The hierarchy of transition metal homeostasis: iron controls manganese accumulation in a unicellular cyanobacterium. *Biochimica et Biophysica Acta (BBA) - Bioenergetics*.

[44] Hernández-Prieto MA, Semeniuk TA, Giner-Lamia J, Futschik ME (2016). The transcriptional landscape of the photosynthetic model cyanobacterium *Synechocystis* sp. PCC6803. *Sci Rep*.

[45] Salomon E, Keren N (2015). Acclimation to environmentally relevant Mn concentrations rescues a cyanobacterium from the detrimental effects of iron limitation. Environ Microbiol.

[46] Lee JW, Helmann JD (2007). Functional specialization within the Fur family of metalloregulators. Biometals.

[47] Ma Z, Faulkner MJ, Helmann JD (2012). Origins of specificity and cross-talk in metal ion sensing by *Bacillus subtilis* Fur. Mol Microbiol.

[48] Howard SP, Herrmann C, Stratilo CW, Braun V (2001). *In vivo* synthesis of the periplasmic domain of TonB inhibits transport through the FecA and FhuA iron siderophore transporters of *Escherichia coli*. J Bacteriol.

[49] Lau CKY, Krewulak KD, Vogel HJ (2016). Bacterial ferrous iron transport: the Feo system. FEMS Microbiol Rev.

[50] Chenault SS, Earhart CF (1992). Identification of hydrophobic proteins FepD and FepG of the *Escherichia coli* ferrienterobactin permease. J Gen Microbiol.

[51] Bosma EF, Rau MH, van Gijtenbeek LA, Siedler S (2021). Regulation and distinct physiological roles of manganese in bacteria. FEMS Microbiol Rev.

[52] Martin JE, Waters LS, Storz G, Imlay JA (2015). The *Escherichia coli* small protein MntS and exporter MntP optimize the intracellular concentration of manganese. PLoS Genet.

[53] Bartsevich VV, Pakrasi HB (1999). Membrane topology of MntB, the transmembrane protein component of an ABC transporter system for manganese in the cyanobacterium *Synechocystis* sp. strain PCC 6803. J Bacteriol.

[54] Imlay JA (2014). The mismetallation of enzymes during oxidative stress. J Biol Chem.

[55] Millaleo R, Reyes-Díaz M, Alberdi M, Ivanov AG, Krol M (2013). Excess manganese differentially inhibits photosystem I versus II in *Arabidopsis thaliana*. J Exp Bot.

[56] Csatorday K, Gombos Z, Szalontai B (1984). Mn and Co toxicity in chlorophyll biosynthesis. Proc Natl Acad Sci U S A.

[57] Pérard J, Ollagnier de Choudens S (2018). Iron-sulfur clusters biogenesis by the SUF machinery: close to the molecular mechanism understanding. J Biol Inorg Chem.

[58] Shen G, Balasubramanian R, Wang T, Wu Y, Hoffart LM (2007). SufR coordinates two [4fe-4S]2+, 1+ clusters and functions as a transcriptional repressor of the sufbcds operon and an autoregulator of sufr in cyanobacteria. J Biol Chem.

[59] Mills LA, McCormick AJ, Lea-Smith DJ (2020). Current knowledge and recent advances in understanding metabolism of the model cyanobacterium *Synechocystis* sp. PCC 6803. Biosci Rep.

