# Supplementary material for: Study of excess manganese stress response highlights the central role of manganese exporter Mnx for holding manganese homeostasis in the cyanobacterium Synechocystis sp. PCC 6803
Source: Microbiology (Reading). 2024 Nov 7;170(11):001515. doi: 10.1099/mic.0.001515 (PMC11649195; doi:10.1099/mic.0.001515)
Supplement: Uncited Supplementary Material 1. [file mic-170-01515-s001.pdf]

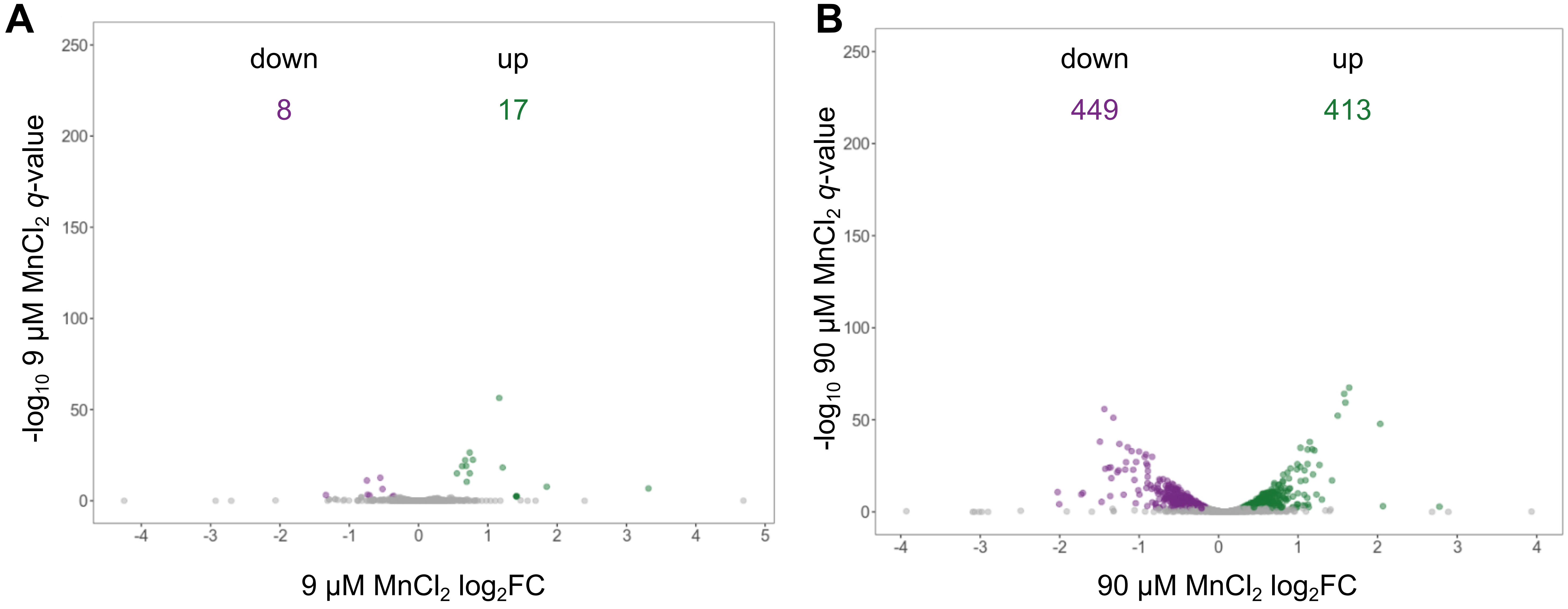

Fig. S1: Volcano plots of the global transcriptome responses under **(A)** control (9 μM $MnCl_2$) and **(B)** Mn excess (90 μM $MnCl_2$) conditions. Shown are log_2-fold changes (log_2FC) of Δ*mnx* mutant line *versus* WT under each condition. Differentially expressed genes ($q < 0.01$; edgeR, [24]) are plotted in green (up) or violet (down) respectively. The number of differentially expressed genes is given in violet for significantly downregulated and green for significantly upregulated genes.

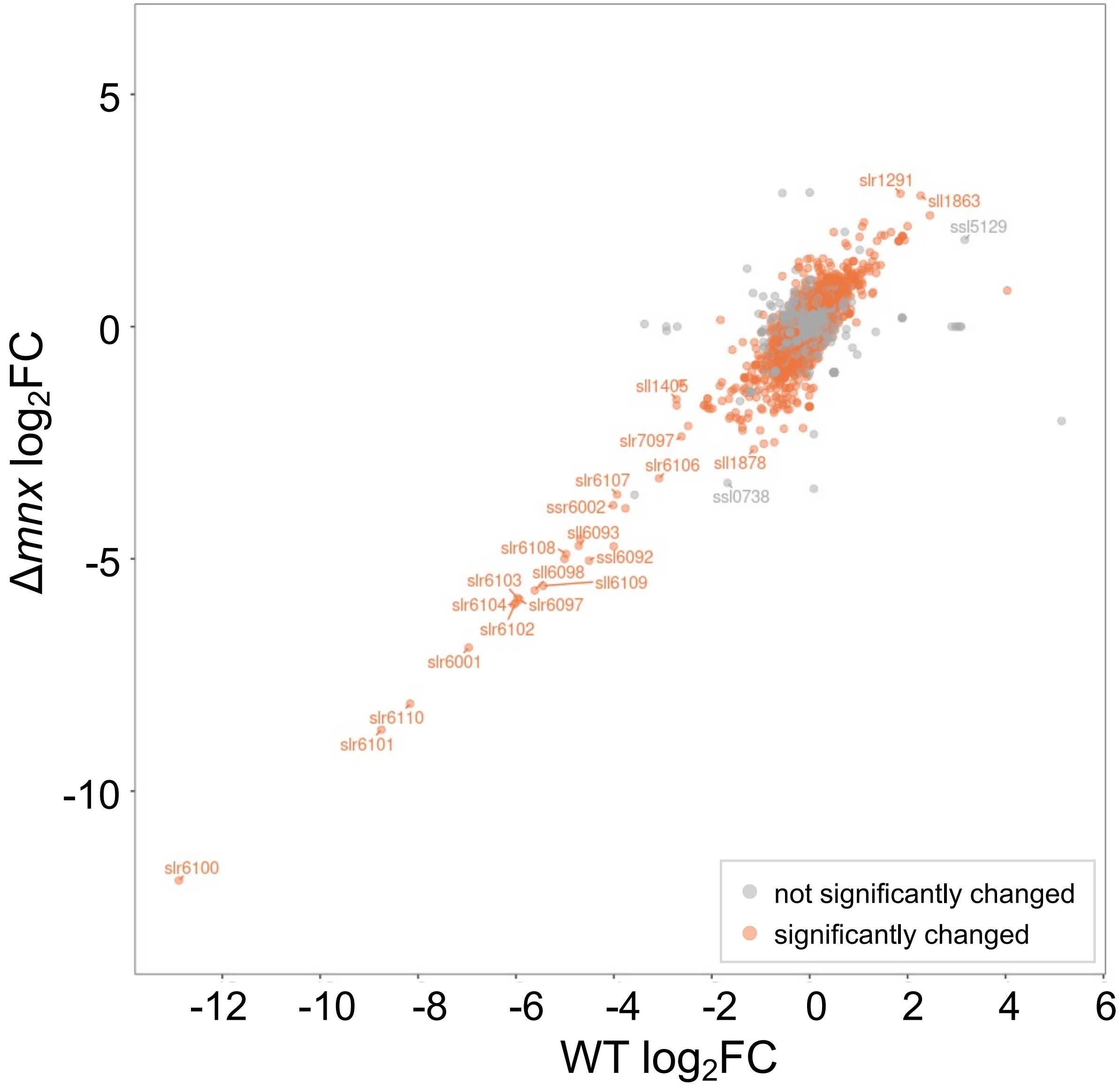

Fig. S2: Scatterplot of the transcript abundances as log_2FC in WT and Δ*mnx*. Orange color indicates significantly different ($q \leq 0.01$) transcript abundances in WT and Δ*mnx* upon $MnCl_2$ treatment, grey represents not significant differences. Gene loci of transcripts with a log_2FC of $\geq |2.5|$ are displayed.
